# P21-activated kinase 5 potentiates the chemoresistant phenotype of liver cancer

**DOI:** 10.1038/s41392-020-00409-y

**Published:** 2021-02-05

**Authors:** Ding-Guo Zhang, Chan-Chan Gong, Xiao-Jin Wu, Xin Ren, Randee S. Sedaka, Wei-Cong Chen, Fu-Chun Huo, Cheng Chen, Wen-Qi Du, Dong-Sheng Pei

**Affiliations:** 1 Department of Pathology, Xuzhou Medical University, Xuzhou, China; 2 Department of Medicine, University of Alabama at Birmingham, Birmingham, AL USA; 3 Department of Radiation Oncology, Xuzhou Municipal Hospital Affiliated to Xuzhou Medical University, Xuzhou, China

**Keywords:** Cancer therapy, Oncogenes

**Dear Editor,**

Systemic chemotherapy is hindered for the treatment of advanced HCC, largely due to the multidrug-resistance phenotype.^1^ One of the major mechanisms of drug resistance, increased drug efflux, is mediated by various membrane transporters. ABCB1 (MDR1) is one of the most extensively studied members of the ATP-binding cassette family and has been shown to be elevated in multiple tumor types.^2^ However, most clinical trials with ABCB1 inhibitors failed to reach the desired endpoints, which calls for an in-depth understanding of ABCB1 gene regulation.

p21-activated kinase 5 (PAK5) possesses oncogenic characteristics, which are largely attributed to its anti-apoptotic and/or pro-invasive roles.^3,4^ Overexpression of PAK5 inhibits cisplatin-induced apoptosis of HCC.^5^ However, it is unclear whether PAK5 regulates membrane transporters responsible for the HCC multidrug-resistant phenotype. In this study, we investigated the mechanisms by which PAK5 enhances HCC chemoresistance following treatment with different anticancer drugs, ultimately revealing the functional and clinical insights of targeting PAK5.

We collected cancerous and corresponding paracancerous tissues from 273 HCC patients for immunohistochemical analysis. Expression of PAK5 and ABCB1 was increased in HCC compared to adjacent tissues (Fig. 1a and Supplementary Fig. S1a). We found that high expression of PAK5 was associated with tumor size, pT status, pN status, TNM stage, and HCC pathological type. High ABCB1 expression was associated with pT status, TNM status, HCC pathological type, capsular presence, and AFP (Supplementary Table S1). Additionally, both PAK5 and ABCB1 expression were negatively correlated with 5-year survival rates (Fig. 1b). Also, we determined that the expression of PAK5 and ABCB1 were positively correlated with each other (Supplementary Table S2).

**Fig. 1:**
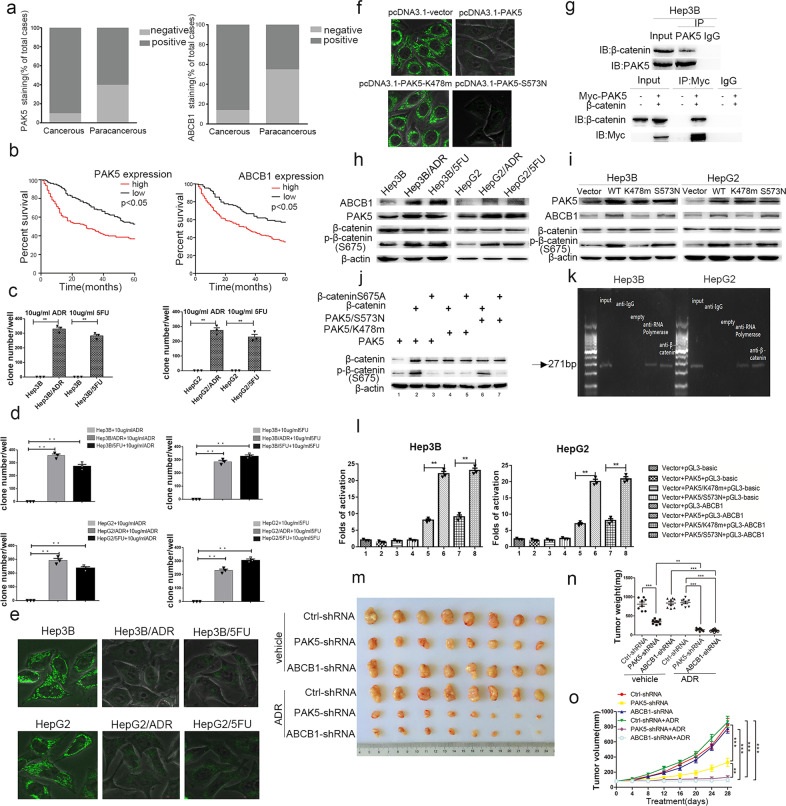
PAK5-mediated phosphorylation and nuclear translocation of β-catenin facilitates ABCB1 regulation and confers the chemoresistant phenotype in HCC. **a** PAK5 and ABCB1 expression was higher in HCC cancerous vs. paracancerous tissues. **b** High PAK5 and ABCB1 expression correlated with worse 5-year survival in HCC patients (*P* < 0.05, log-rank test). **c** Clone formation was elevated in Hep3B- and HepG2-resistant cells. **d** Colony formation following ADR and 5FU treatment in resistant Hep3B and HepG2 cells was elevated. **e** Rhodamine staining was decreased in ADR/5FU-resistant cells and (**f**) in Hep3B cells transfected with PAK5, or S573N-PAK5. **g** Endogenously and exogenously expressed PAK5 immunoprecipitated with β-catenin in Hep3B cells. **h** ABCB1, PAK5, and phosphorylated β-catenin expression were increased in resistant cell lines. **i** PAK5, ABCB1, and phosphorylated β-catenin expression were increased in both Hep3B and HepG2 cells transfected with PAK5 or S573N-PAK5. **j** Cells co-transfected with PAK5/β-catenin or S573N-PAK5/β-catenin displayed enhanced β-catenin phosphorylation. **k** ChIP analysis showed an abundance of β-catenin on the ABCB1 promoter in parental cells. **l** Luciferase reporter activity was increased in cells transfected with PAK5 or S573N-PAK5. **m** Representative images of subcutaneous xenograft tumors formed by parental or ADR-resistant Hep3B cells in nude mice treated with or without ADR for four weeks. **n**, **o** Tumor weight and volume were decreased in PAK5-silenced and resistant cells. **P < 0.01, ***P < 0.001

We established the following resistant cell strains: HepG2, Hep3B resistant to adriamycin (HepG2/ADR, Hep3B/ADR); HepG2, Hep3B resistant to 5-fluorouracil (HepG2/5FU, Hep3B/5FU). We found greater cell proliferation in resistant strains in the presence of ADR or 5FU, compared to parental cells (Supplementary Fig. S1b, c). Results from a clone-forming assay showed that the drug-resistant strains formed a large number of cell colonies in the presence of ADR or 5FU, while none were formed in the parental cells (Fig. 1c and Supplementary Fig. S5a). We found decreased cell apoptosis in resistant strains after treatment of ADR or 5FU, compared to parental cells (Supplementary Fig. S1d, e). These results demonstrate that the resistant cells exhibit characteristics of a drug-resistant phenotype.

Following ADR treatment, the proliferating abilities of Hep3B/5FU and HepG2/5FU cells were significantly increased compared to the parental cells (Supplementary Fig. S2a, b). Similar effects were observed in Hep3B/ADR and HepG2/ADR cells treated with 5-fluorouracil (Supplementary Fig. S2a, b). Also, we observed that Hep3B/ADR and Hep3B/5FU and HepG2/ADR and HepG2/5FU (Fig. 1d and Supplementary Fig. S5b) cells formed more clones than corresponding parental cells. These results indicate that singly resistant Hep3B and HepG2 HCC cell strains may display multidrug resistance. Next, we found that the fluorescent intensity in resistant cells was weaker than that of the parental cells by rhodamine staining (Fig. 1e). To investigate the role of PAK5 on drug-resistant protein expression, we transfected Hep3B cells with vector, PAK5, PAK5-K478m (constitutively inactive), or PAK5-S573N (constitutively activate). The fluorescent intensity in PAK5 and PAK5-S573N-transfected cells was weaker than that of vector and PAK5-K478m transfected cells (Fig. 1f), suggesting that PAK5 activation may be involved in the regulation of drug-resistant proteins. To determine the role of ABCB1 in drug resistance, we performed western blot experiments on parental and resistant cells (Supplementary Fig. S2c) and found that the expression of ABCB1 was significantly increased in drug-resistant strains.

We next wanted to uncover the potential of PAK5 to regulate pivotal pathways in the development of HCC. Immunoprecipitation experiments showed an interaction between endogenous PAK5 and β-catenin proteins as well as exogenous PAK5 and β-catenin proteins when transfected into parental cells (Fig. 1g). Interestingly, this interaction increased in resistant cells compared with parental cells (Supplementary Fig. S3a). Western blot analysis showed increased expression of PAK5 and ABCB1 in resistant cells (Fig. 1h). We hypothesized that PAK5 affects the phosphorylation of β-catenin. We scanned the protein sequence of β-catenin and identified S675 (activation site) as a potential target site for PAK5. Expression of phosphorylated-β-catenin was increased in resistant cells compared with parental cells. Hep3B and HepG2 cells transfected with PAK5 and PAK5-S573N plasmids displayed increases in total protein expression of ABCB1 and phosphorylated-β-catenin (S675) (Fig. 1i). We observed an increase in phosphorylated-β-catenin (S675) expression in cells co-transfected with PAK5/β-catenin and S573N-PAK5/β-catenin (lanes 2 and 6) (Fig. 1j). These results suggest that PAK5 could bind with β-catenin and affect its phosphorylation status.

Next, we determined the subcellular distribution of phosphorylated β-catenin. Parental cells transfected with exogenous PAK5 or PAK5-S573N displayed decreased expression of total and phosphorylated-β-catenin in the cytoplasm and increased in the nucleus (Supplementary Fig. S3b). The expression of both total and phosphorylated-β-catenin (S675) was decreased in the cytoplasm and increased in the nucleus of drug-resistant strains compared with parental cells (Supplementary Fig. S3c). Meanwhile, expression of PAK5 was increased in both the cytoplasm and nucleus. Moreover, we observed increased nuclear β-catenin staining in resistant strains compared to parental cells (Supplementary Fig. S3d) as well as in the nuclei of PAK5 and S573N-transfected cells (Supplementary Fig. S3e).

Then, we sought to elucidate whether PAK5-mediated phosphorylation of β-catenin is involved in the upregulation of ABCB1. We found that β-catenin binds to the ABCB1 promoter in both Hep3B and HepG2 cells (Fig. 1k), and that this binding was increased in the resistant cells compared with parental cells (Supplementary Fig. S4a). Subsequently, we found that β-catenin binding to ABCB1 was increased in the PAK5 and PAK5-S573N groups compared with vector and PAK5-K478m (Supplementary Fig. S4b). However, ABCB1 was not affected by PAK5 when β-catenin is knocked down (Supplementary Fig. S4c). Moreover, parental cells expressing a luciferase-tagged ABCB1 promoter and co-transfected with PAK5 and β-catenin plasmids had significantly increased luciferase activity (Fig. 1l and Supplementary Fig. S4d), suggesting that PAK5 regulates ABCB1 promoter activity. These results indicate that PAK5-mediated β-catenin phosphorylation promotes the transcriptional activity of ABCB1.

To validate the results obtained from HCC cells in vitro, we conducted in vivo experiments in nude mice. Mice were treated with ADR or saline with simultaneous subcutaneous lentiviral injections of HepG2/ADR loads (Ctrl-shRNA, PAK5-shRNA, or ABCB1-shRNA). Tumor weight and volume were measured at the end of the study. Significant inhibition of tumor growth was not observed in mice after ADR treatment with Ctrl-shRNA (Fig. 1m–o). Tumor growth was significantly inhibited in mice transfected with PAK5-shRNA or ABCB1-shRNA. Immunohistochemical staining of tumors showed that low PAK5 expression reduced ABCB1 expression (Supplementary Fig. S4e). The expressions of PAK5 and ABCB1 in mouse tumor tissues were also examined by western blot (Supplementary Fig. S4f). Furthermore, we found that the phosphorylation status and localization of β-catenin in the tumor tissues verified the in vitro data shown prior (Supplementary Fig. S4g, h).

Our previous studies support the idea that downstream targets of PAK5 form a complex web and that PAK5-mediated regulation of cancer initiation and progression is multifactorial and dependent on cancer type^3–5^. Future efforts will be made to determine the mechanism(s) underlying the overexpression of PAK5 in resistant strains. We have known that DNA methylation plays a key role in modifying genetic information during cancer initiation and progression and that alterations in methylation could precede cancer formation. It will be interesting to know whether reversing PAK5 methylation by targeting maintenance DNA methylation machinery could rescue the chemoresistant phenotype of HCC. In summary, our study establishes that PAK5-mediated phosphorylation and nuclear translocation of β-catenin facilitates ABCB1 regulation and confers the chemoresistant phenotype in HCC.

## Supplementary information

Supplementary materials

Supplementary Fig. S1

Supplementary Fig. S2

Supplementary Fig. S3

Supplementary Fig. S4

Supplementary Fig. S5

## Data Availability

All datasets on which the conclusions of the paper rely are presented in the paper.
