# Supplementary material for: P21-activated kinase 5 potentiates the chemoresistant phenotype of liver cancer
Source: Signal Transduct Target Ther. 2021 Feb 5;6:47. doi: 10.1038/s41392-020-00409-y (PMC7862393; doi:10.1038/s41392-020-00409-y)
Supplement: Supplementary file 1 — Supplementary materials [file 41392_2020_409_MOESM1_ESM.docx]

Supplementary Materials for

**P21-activated kinase 5 potentiates the chemoresistant phenotype of liver cancer**

Ding-Guo Zhang^1,2#^, Chan-Chan Gong^1#^, Xiao-Jin Wu^3#^, Xin Ren^1^, Randee S. Sedaka^2^, Wei-Cong Chen^1^, Fu-Chun Huo^1^, Cheng Chen^1^, Wen-Qi Du^4^, Dong-Sheng Pei^1^*

**This file includes:**Materials and Methods
References for “Material and Methods” section
Figures. S1 to S5
Table S1, 2

**Materials and Methods**

**Cell lines and generation of chemoresistant cancer cells**

Human HCC cell lines Hep3B and HepG2 were obtained from the Shanghai Institute of Biochemistry and Cell Biology, Chinese Academy of Sciences (Shanghai, China). Hep3B were cultured in RPMI-1640 (Gibco, Shanghai, China) and HepG2 were cultured in DMEM (Gibco, Grand Island, NY, USA), both supplemented with 10% fetal calf serum and grown in a 37 °C humidified incubator.

Hep3B and HepG2 5FU-resistant cell lines were selected based on constant exposure of the parental cells to an incremental increase of 5FU. Briefly, through increasing selection pressure in liquid culture, surviving cells which became resistant to 5FU were picked and transferred to medium containing increasing concentrations of 5FU. After establishment, resistant lines were continuously cultured in the presence of 5FU. Hep3B and HepG2 ADR-resistant cell lines were undertaken using a previously describe method ^1^.

**Transfection**

Non-specific control siRNA, PAK5 siRNA, ABCB1 siRNA and β-catenin siRNA were designed and synthesized by GenePharma (Shanghai, China). The sequences of siRNAs are as follows: PAK5, 5′-CAAAGTCTTCGTACCTGAATC-3′; ABCB1, 5′-AGAAACCAACTGTCAGTGTAT-3′; β-catenin, 5′-AAACTACTGTGGACCACAAGCCCTGTCTC-3′. Cells were transfected with siRNA control (siCtrl), siRNA PAK5 (siPAK5), siRNA ABCB1 (siABCB1) and siRNA β-catenin (siβ-catenin) using siLentFect™ Lipid Reagent (Bio-Rad, Hercules, CA, USA) following the manufacturer’s protocol. GenePharma pcDNA3.1-control, pcDNA3.1-PAK5, pcDNA3.1-K478m-PAK5, pcDNA3.1-S573N-PAK5, pcDNA3.1-β-catenin and pcDNA3.1-Ser675A-β-catenin were transiently transfected into cells using the X-tremeGENE HP DNA Transfection Reagent (Roche, Indianapolis, IN, USA). Cells were harvested after 24-48 h of transfection.

**Antibodies**

Antibodies against the following proteins were used: PAK5, ABCB1 (Abcam); MRP2, β-catenin, c-Myc (Santa Cruz); p-β-catenin(S675) (Cell Signaling Technology); β-actin (Zhongshan biotech); ABCG2, LRP (Proteintech); rabbit HRP, mouse HRP (Vicmed).

**Western blotting**

Cells and tissues were harvested using RIPA lysis buffer (Beyotime). Lysates were pelleted by centrifugation at 12,000xg for 15 min at 4 °C and the protein concentrations were determined using the bicinchoninic acid kit (BCA, Pierce, USA) according to the manufacturer’s instructions. Proteins were separated by SDS-polyacrylamide gel electrophoresis, transferred to a nitrocellulose membrane, blocked in 5% bovine serum albumin for 2 h, then incubated overnight at 4 °C with the indicated primary antibodies. Membranes were washed for 15 min, probed with HRP-conjugated secondary antibodies, and imaged using the Chemistar™ High-sig ECL Western Blot Substrate (Tanon, shanghai, China).

**Co-immunoprecipitation (co-IP)**

Cells were transfected with pcDNA3.1-Myc (control) plasmids, pcDNA3.1-Myc-PAK5 plasmids and pcDNA3.1-β-catenin plasmids and harvested in lysis buffer containing a cocktail of protease and phosphatase inhibitors (Sigma Aldrich). Cell lysates (1000 μg) were incubated with primary antibody at 4 °C overnight, then 30 μl of Protein A/G Sepharose beads (Santa Cruz) were added for another 4-6 h. The immunoprecipitates were washed and analyzed by western blot.

**Patients and specimens**

Hepatocellular carcinoma tissues and adjacent, non-cancerous tissues (at least 2 cm away from the tumor) were collected from 273 patients from the Affiliated Hospital of Xuzhou Medical University. These specimen were used for tissue microarray construction and diagnoses were independently confirmed by two pathologists. The histological grade was classified into I to IV according to criteria set by the World Health Organization. All patients signed informed consent documents and were termly followed for four to eighty months to evaluate post-operative survival.

**Immunohistochemical** **staining** **and evaluation**

Immunohistochemistry was performed with a Streptavidin-peroxidase (Sp) Kit (Zhongshan biotech, Beijing, China). Tissue microarray (TMA) slides were dewaxed at 65 °C for 1 h followed by two 20 min washes with xylene and then rehydrated with different concentrations of ethanol, in sequence, and finally with distilled water for 2 min each. The slides were then put into 95°C citrate buffer for 3 min, endogenous peroxidase activity was blocked with 3% hydrogen peroxide, and later blocked with 5% normal goat serum to block non-specific reactions for 35 min. The slides were incubated with primary antibodies overnight at 4 °C. The sections were then incubated with a horseradish peroxidase-conjugated secondary antibody, followed by peroxidase substrate DAB kit (DAB; Zhongshan biotech) to develop brown color. After hematoxylin counterstain and dehydration, the sections were sealed with cover slips. Staining was assessed by two independent observers using light microscopy (Olympus BX-51 light microscope) and captured by a Camedia Master C-3040 digital camera.

Positive PAK5 immunostaining was graded according to both the intensity and percentage of cells with positive staining. Intensity was scored 0–3 (0 = negative; 1 = weak; 2 = moderate; 3 = strong), while percentage of positive cells was scored into four categories: 1 (0–25%), 2 (26–50%), 3 (51–75%) and 4 (76–100%). PAK5 staining was evaluated by the immunoreactive score (IRS), which is calculated by multiplying the staining intensity score by the percentage of positive cells. Positive ABCB1 immunostaining was undertaken using a previously describe method^2^.

**Cell proliferation assay**

Cell proliferation was measured via the Cell Counting Kit-8 (CCK-8) kit (Vicmed, Xuzhou, China). Briefly, cells were plated in a 96-well microplate (Corning) and incubated at 37 °C with 5% CO2. CCK-8 reagent (10 μl) solution with 100 μl serum-free medium was added to each well at 24, 48, 72, and 96 h, respectively, followed by incubation for 2 h at 37 °C with 5% CO2. Absorbance at 450 nm was measured by a multi-function, enzyme-linked analyzer (Biotek Instruments, Winooski, VT, USA).

**Rhodamine staining**

Transfected Hep3B, Hep3B/ADR, Hep3B/5FU, HepG2, HepG2/ADR, and HepG2/5FU cells were cultured in 6-well plates and incubated with 0.2µg/ml rhodamine (Solarbio) at 37°C and 5% CO2 for 30 minutes. Afterwards, cells were washed with PBS for 15 minutes before being imaged using immunofluorescent confocal laser scanning microscopy (Zeiss LSM 880).

**Immunofluorescence and confocal microscopy**

Transfected Hep3B, Hep3B/ADR, Hep3B/5FU, HepG2, HepG2/ADR, and HepG2/5FU cells were cultured in 6-well plates. Cells were fixed in 4% paraformaldehyde for 20 min at room temperature, washed with PBS for 15 min, permeabilized with 0.5% Triton for 15 min, then blocked with PBS containing 5% BSA for 30 min. Subsequently, cells were incubated with PAK5 (rabbit anti-human, 1:200; Abcam) and β-catenin (mouse anti-human, 1:200; Santa Cruz) antibodies diluted in blocking buffer overnight at 4 °C. This was followed by secondary antibody staining with coraLite594**-**conjugated goat anti-rabbit IgG(H+L) and coraLite488-conjugated affinipure goat anti-mouse IgG(H+L) diluted 1:100 in blocking buffer for 30 min. Nuclei were stained with 4′, 6-Diamidino-2-phenylindole (DAPI) for 10 min. Images were captured by immunofluorescent confocal laser scanning microscopy (Zeiss LSM 880).

**Colony formation assay**

Cells were cultured in 6-well plates at a density of 200 cells/well. After a two-week incubation, cells were washed with PBS, allowed to air dry at room temperature, and colony number was counted. Each experiment was performed in triplicate.

**Flow cytometry analysis**

Cell apoptosis was determined by flow cytometry using FITC Annexin V Apoptosis Detection Kit (BD Pharmingen). Briefly, 2 × 10^6^ cells were washed twice with PBS and diluted in 100 μl of BD kit (1×Binding Buffer) containing 5 μl FITC Annexin V and PI for 15 min at room temperature in the dark. After incubation, the population of Annexin V-positive cells was analyzed by a FACS Aria cytometer (Becton cytomics FC500).

**Luciferase reporter assay**

The ABCB1 promoter was subcloned into a pGL3-basic luciferase expression vector (Genepharma). Cells were split into 24-well plates and co-transfected with luciferase vector pGL3-basic (control) or pGL3-ABCB1, together with pcDNA3.1 (control), pcDNA3.1-PAK5, pcDNA3.1-K478m-PAK5, pcDNA3.1-S573N-PAK5, pcDNA3.1-β-catenin, or pcDNA3.1-S675A-β-catenin plasmids. After 24 h, whole-cell lysates were collected and luciferase activity was calculated by the Luciferase Reporter System (Promega, Shanghai, China) according to the manufacturer’s protocol. Each experiment was performed in triplicate.

**Chromatin immunoprecipitation (ChIP)**

ChIP-PCR assays were carried out on 5×10^6^ cells/well and prepared with the ChIP assay kit (Merck Millipore). DNA samples (2 μl) were then amplifed by PCR using ABCB1 promoter primer pairs (forward: 5′-AGAAGCCATCACCTCCAGGA-3′; reverse: 5′-AATTGCTGTCTTGAAGGATATACATTTACA-3′). The resulting precipitated DNA was analyzed by PCR as described previously.

**Tumorigenicity *in vivo***

Thirty 4-week-old female BALB/cA-nude mice were purchased from Beijing Huafukang Bioscience (Beijing, China). Stable Hep3B (Ctrl-shRNA, PAK5-shRNA and ABCB1-shRNA) and Hep3B/ADR (Ctrl-shRNA, PAK5-shRNA and ABCB1-shRNA) cells were concentrated to 2 × 10^6^/100 μl PBS and subcutaneously injected into mice. After 4 weeks, the mice were sacrificed by cervical decapitation to harvest tumors and measure tumor weights.

**Ethics statement**

All experiments involving human participants were approved by the Review Board of the Affiliated Hospital of Xuzhou Medical University. All patients provided a written informed consent prior to the study. Animal experiments were performed in strict accordance with the protocols approved by the Institutional Animal Care and Use Committee of Xuzhou Medical University.

**Statistical analysis**

All statistical analyses were conducted by SPSS version 16.0 (SPSS Inc., Chicago, IL, USA). Statistical significance of Student’s t-test was presented for two-group comparisons. Quantitative data were determined as means ± SD. The χ2 test was used to analyze the correlation between PAK5/ABCB1 expression and the clinico-pathological parameters of HCC patients. Survival analysis was estimated by the Kaplan–Meier method and the log-rank test. The correlation analysis between PAK5 and ABCB1 was evaluated by spearman test. *P* < 0.05 was considered significant.

**References**

1. Huang M, *et al.* Targeting KDM1A attenuates Wnt/beta-catenin signaling pathway to eliminate sorafenib-resistant stem-like cells in hepatocellular carcinoma. *Cancer lett* **398**, 12-21 (2017).

2. Guo. H, *et al.* Down-Regulation of the Cyclin Dependent Kinase Inhibitor p57 Is Mediated by Jab1/Csn5 in Hepatocarcinogenesis. *Hepatology*, **63**, 891-913 (2016).

**Figure S1**


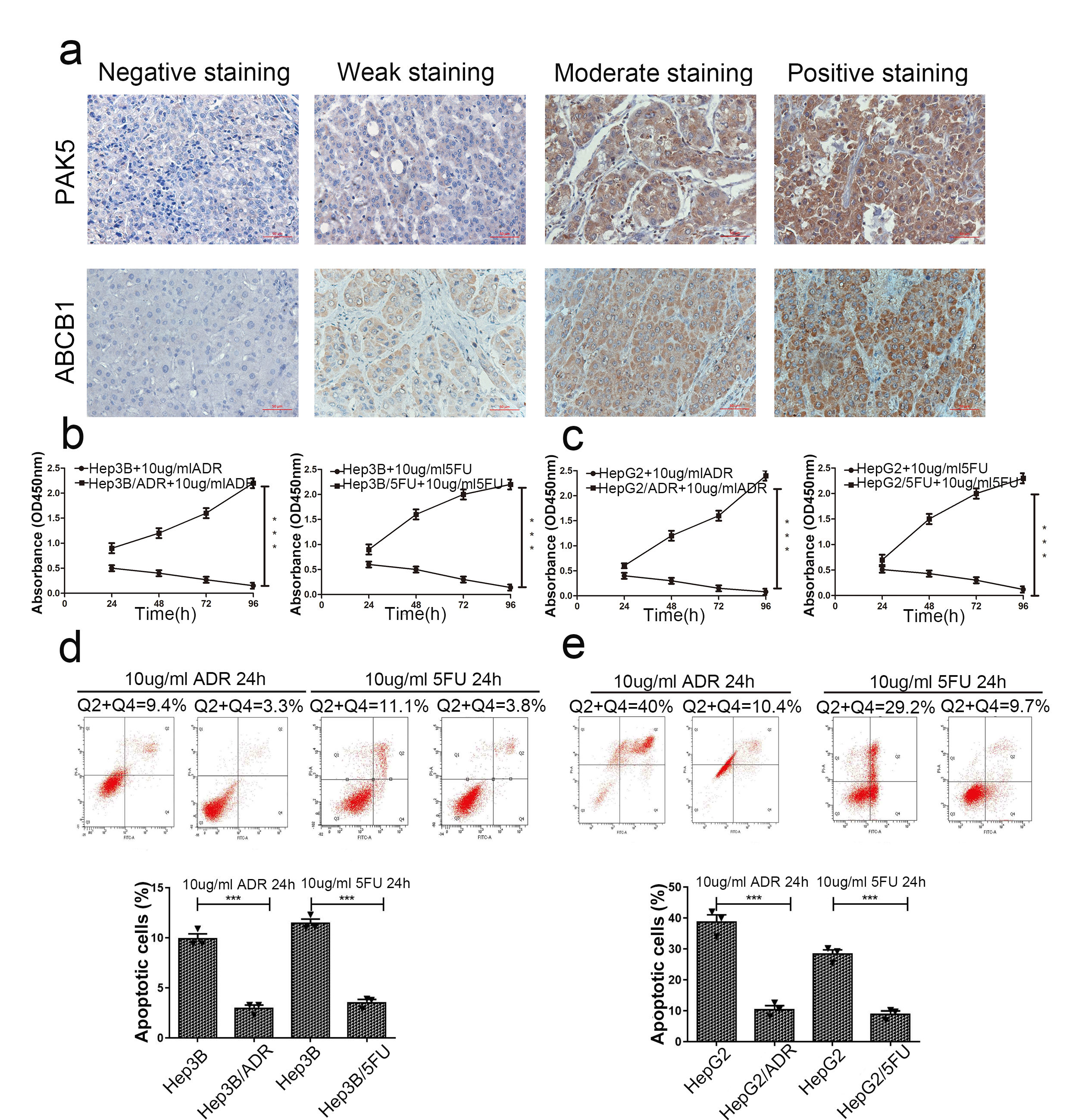


**Figure. S1. PAK5/ABCB1 immunohistochemical staining in HCC patients and establishment of resistant HCC cells.** (**a**) Representative images of PAK5/ABCB1 immunohistochemical staining in HCC patients (400X). (**b**) Cell proliferation was increased in Hep3B and (**c**) HepG2 resistant cells compared to parental cells. (**d**) Apoptosis was reduced in resistant Hep3B and (**e**) HepG2 cells compared to parental lines. **, *P* < 0.01

**Figure S2**


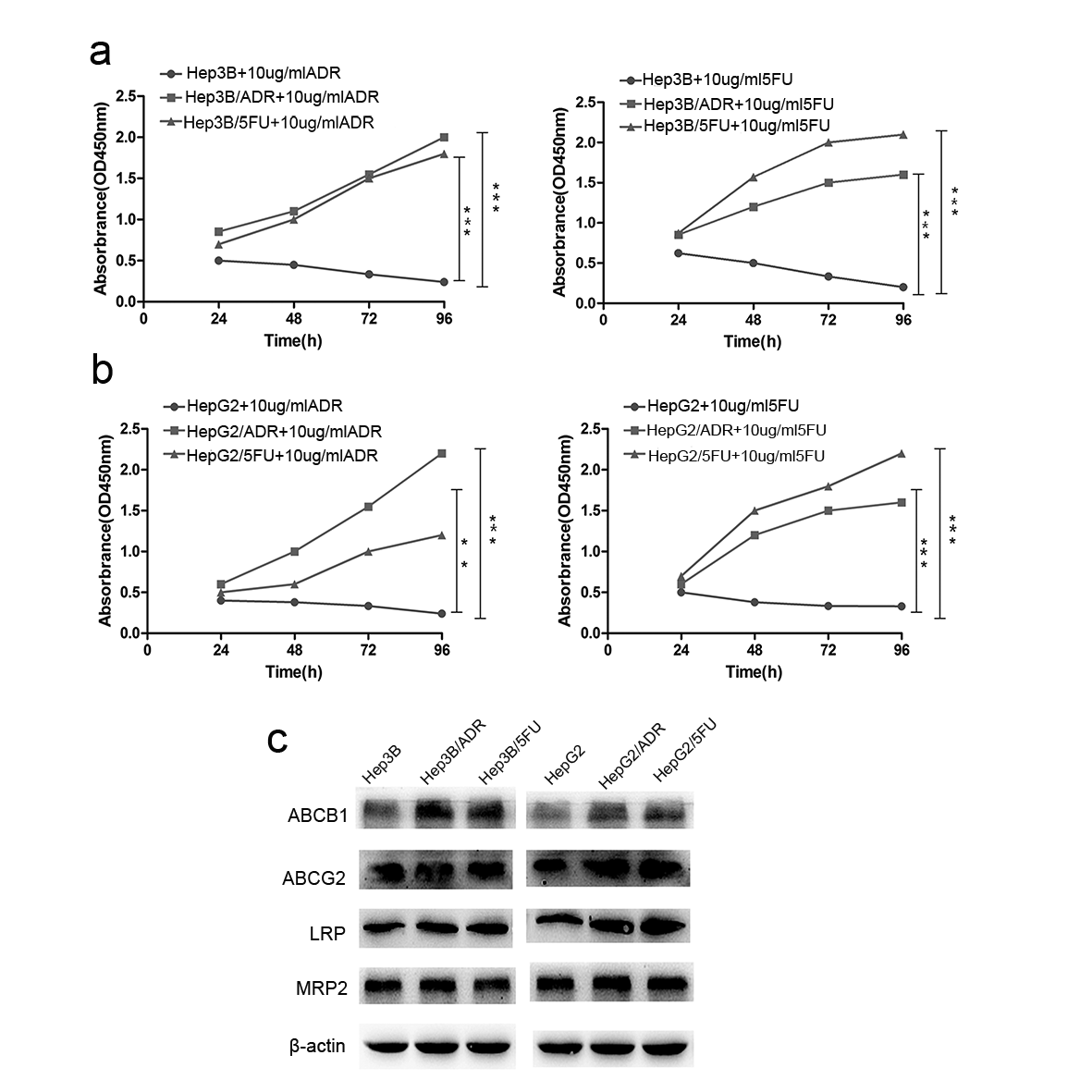


**Figure. S2. Resistant cells exhibit multiple drug resistance compared to parental cells.** (**a**) Cell proliferation was increased following ADR and 5-FU treatment in resistant Hep3B and (**b**) HepG2 cells. **(c)** Expression of ABCB1 was increased in resistant cell lines. **, *P* < 0.01, ***, *P* < 0.001

**Figure S3**


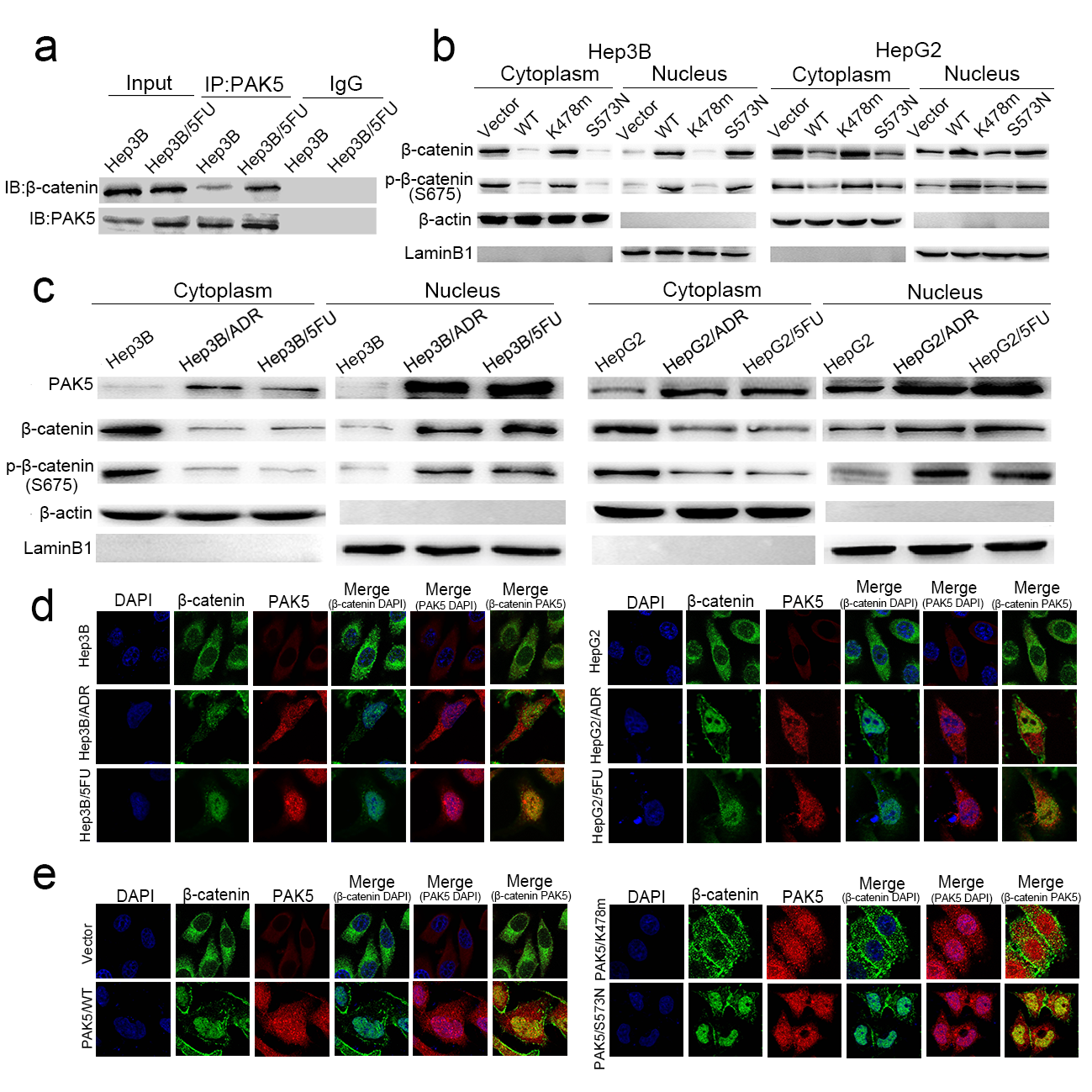


**Figure. S3. PAK5 phosphorylates β-catenin and activates the nuclear translocation of the β-catenin.** (**a**) PAK5 immunoprecipitated with β-catenin to a higher degree in Hep3B/5FU resistant cells. (**b**) PAK5 expression is increased in resistant strains in both the cytoplasm and nucleus, whereas β-catenin and phosphorylated-β-catenin expression is decreased in the cytoplasm and increased in the nucleus of resistant cells. (**c**) Total and phosphorylated β-catenin expression is decreased in cytoplasmic and increased in nuclear fractions of PAK5 and S573N-PAK5 cells. (**d**) Resistant Hep3B and HepG2 cells displayed increased nuclear β-catenin and PAK5 staining. (**e**) PAK5 and PAK5-S573N transfected cells expressed more nuclear β-catenin and PAK5. β-catenin (green), PAK5 (red), and nuclei (blue), 400X.

**Figure S4**


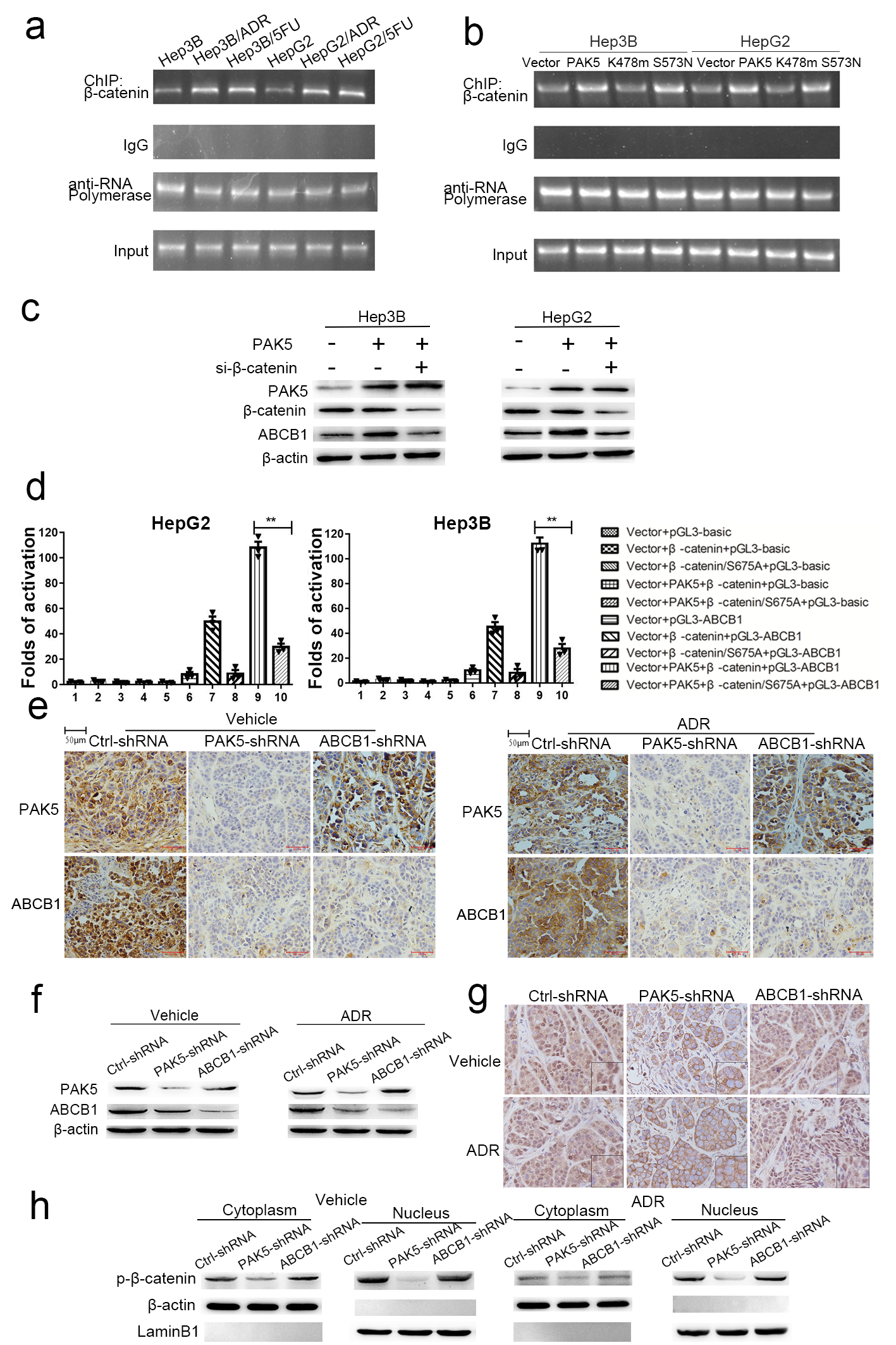


**Figure. S4. β-catenin targets the ABCB1 promoter to active ABCB1 transcription and xenograft staining of PAK5/ABCB1.** (**a**) Binding of β-catenin to ABCB1 was elevated in ADR/5FU resistant cells as well as in (**b**) cells transfected with PAK5 or S573N-PAK5. (**c**) ABCB1 was not affected by PAK5 when β-catenin is knocked down. (**d**) Increased luciferase reporter activity in cells co-transfected with PAK5 and β-catenin. (**e**) Representative xenograft staining showed decreased PAK5 in PAK5-silenced, but not ABCB1-silenced cells, whereas ABCB1 staining was decreased in both PAK5- and ABCB1-silenced cells regardless drug treatment (400X). (**f**) Knockdown of PAK5 expression reduced ABCB1 expression in mouse tumor tissues. (**g**) Decreased PAK5 shifts localization of β-catenin from nuclear to cytoplasm in tumor tissues. (**h**) The phosphorylation status of β-catenin in tumor tissues. **, P < 0.01

**Figure S5**


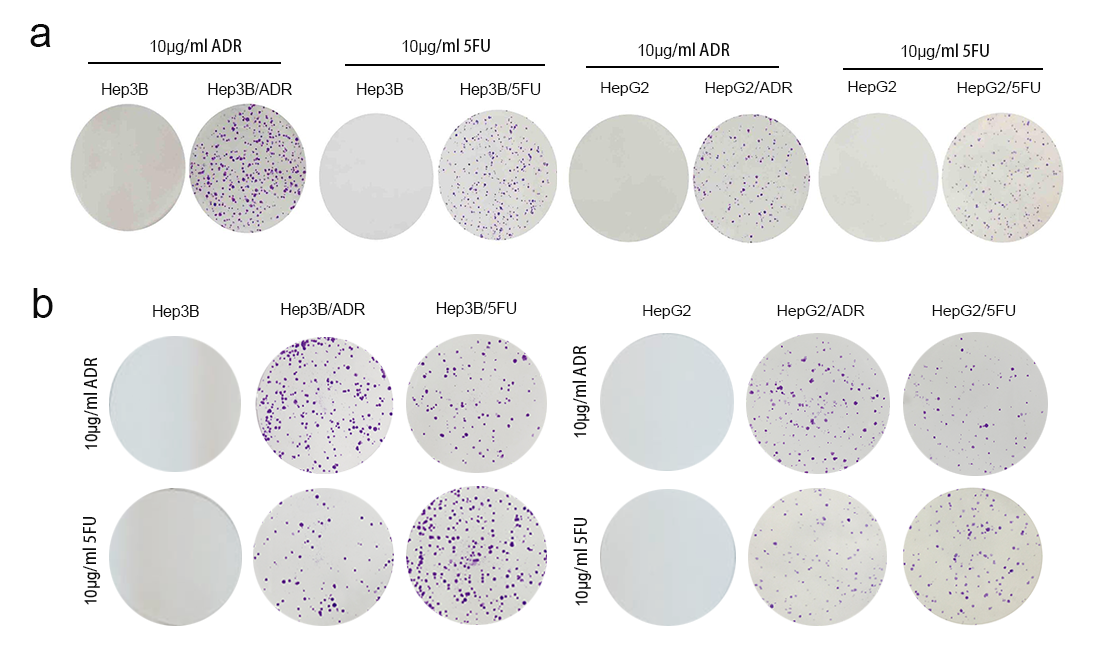


**Figure. S5. Representative images of clone formation assays.** (**a**) Clone formation was elevated in Hep3B and HepG2 resistant cells. (**b**) Colony formation was elevated following ADR and 5-FU treatment in resistant Hep3B and HepG2 cells.

**Table S1** **Correlation of PAK5 and ABCB1 expression with clinicopathologic parameters in HCC**

| **Variables** | **PAK5 staining** | | | | | **ABCB1 staining** | | | | | |
| --- | --- | --- | --- | --- | --- | --- | --- | --- | --- | --- | --- |
|  | **Low(%)** | **High(%)** | **Total** | ***P**** |  |  | **Low(%)** | | **High(%)** | **Total** | ***P**** |
| **Age** |  |  |  |  | |  | |  | |  |  |
| <60 year  ≥60 year | 87(43.9)  40(53.3) | 111(56.1)  35(46.7) | 198  75 | 0.165 | | 83(41.4)  26(36.0) | | 115(58.6)  49(64.0) | | 198  75 | 0.275 |
| **Gender** |  |  |  |  | |  | |  | |  |  |
| Male  Female | 103(45.4)  24(52.2) | 124(54.6)  22(47.8) | 227  46 | 0.399 | | 90(39.6)  19(41.3) | | 137(60.4)  27(58.7) | | 227  46 | 0.834 |
| **Tumor size** |  |  |  |  | |  | |  | |  |  |
| <7cm  ≥7cm | 91(53.2)  30(34.1) | 80(46.8)  58(65.9) | 171  88 | 0.030 | | 80(45.0)  29(31.8) | | 91(54.0)  59(68.2) | | 171  88 | 0.033 |
| **pT status** |  |  |  |  | |  | |  | |  |  |
| pT1  pT2  pT3  pT4 | 80(61.1)  32(41.6)  12(28.6)  2(13.3) | 51(38.9)  45(58.4)  30(71.4)  13(86.7) | 131  77  42  15 | 0.000 | | 70(51.9)  27(32.5)  11(26.2)  1(6.7) | | 65(48.1)  50(67.5)  30(73.8)  14(93.3) | | 131  77  42  15 | 0.000 |
| **pN status** |  |  |  |  | |  | |  | |  |  |
| pN0  pN1 | 126(48.8)  0(00.0) | 132(51.2)  6(100.0) | 258  6 | 0.030 | | 105(40.7)  1(16.7) | | 154(59.3)  5(83.3) | | 258  6 | 0.230 |
| **pM status** |  |  |  |  | |  | |  | |  |  |
| pM0  pM1 | 116(46.4)  0(0.0) | 134(53.6)  4(100.0) | 250  4 | 0.249 | | 104(41.6)  1(25.0) | | 156 (58.4)  3(75.0) | | 250  4 | 0.478 |
| **TNM stage** |  |  |  |  | |  | |  | |  |  |
| Ⅰ  Ⅱ  Ⅲ-Ⅳ | 80(63.6)  22(51.9)  21(36.2) | 49(36.4)  45(48.1)  37(63.8) | 129  77  58 | 0.000 | | 70(52.7)  27(32.5)  12(20.7) | | 59(47.3)  50(67.5)  39(79.3) | | 129  77  58 | 0.000 |
| **HBV infection** |  |  |  |  | |  | |  | |  |  |
| Yes  No | 103(46.0)  24(49.0) | 121(54.0)  25(51.0) | 224  49 | 0.703 | | 91(42.9)  18(40.1) | | 133(57.1)  31(59.2) | | 224  49 | 0.614 |
| **Gross pathologic types** |  |  |  |  | |  | |  | |  |  |
| Nodular  Massive  Diffuse | 90(50.3)  24(30.4)  1(25.0) | 89(49.7)  55(69.6)  3(75.0) | 179  79  4 | 0.009 | | 76(42.4)  16(20.3)  1(25.0) | | 103(57.5)  63(79.7)  3(75.0) | | 179  79  4 | 0.002 |
| **Liver cirrhosis** |  |  |  |  | |  | |  | |  |  |
| Yes  No | 86(50.0)  41(40.6) | 86(50.0)  60(59.4) | 172  101 | 0.133 | | 70(40.7)  36(35.6) | | 102(59.3)  65(64.4) | | 172  101 | 0.408 |
| **With capsular** |  |  |  |  | |  | |  | |  |  |
| Yes  No | 77(55.0)  40(44.4) | 63(45.0)  50(55.6) | 140  90 | 0.118 | | 75(53.6)  32(35.6) | | 65(46.4)  58(64.4) | | 140  90 | 0.008 |
| **Number of tumors** |  |  |  |  | |  | |  | |  |  |
| =1  >1 | 106(52.5)  19(38.8) | 96(47.5)  30(61.2) | 202  49 | 0.085 | | 86(42.6)  18(46.7) | | 116(57.4)  31(63.3) | | 202  49 | 0.457 |
| **AFP**(ng/ml) |  |  |  |  | |  | |  | |  |  |
| <400  ≥400 | 50(37.3)  62(47.3) | 84(62.7)  69(52.7) | 134  131 | 0.099 | | 41(30.6)  68(51.9) | | 93(69.4)  63(48.1) | | 134  131 | 0.000 |
| **Recurrence** |  |  |  |  | |  | |  | |  |  |
| Yes  NO | 87(49.2)  32(40.5) | 90(50.8)  47(59.5) | 177  79 | 0.200 | | 69(39.0)  37(46.8) | | 108(61.0)  42(53.2) | | 177  79 | 0.239 |

**Table S2. Spearman’s rank correlation between PAK5 and ABCB1 expression in HCC**

| ABCB1 | PAK5 | | | spearman | | |
| --- | --- | --- | --- | --- | --- | --- |
|  | Low | High |  |  | Rho | *P* value |
| Low | 65 | 44 | | 0.214 | | 0.000 |
| High | 62 | 102 | |  | |  |
| Total | 127 | 146 | |  | |  |
