# Supplementary figures and images for: P21-activated kinase 5 potentiates the chemoresistant phenotype of liver cancer
Source: Signal Transduct Target Ther. 2021 Feb 5;6:47. doi: 10.1038/s41392-020-00409-y (PMC7862393; doi:10.1038/s41392-020-00409-y)

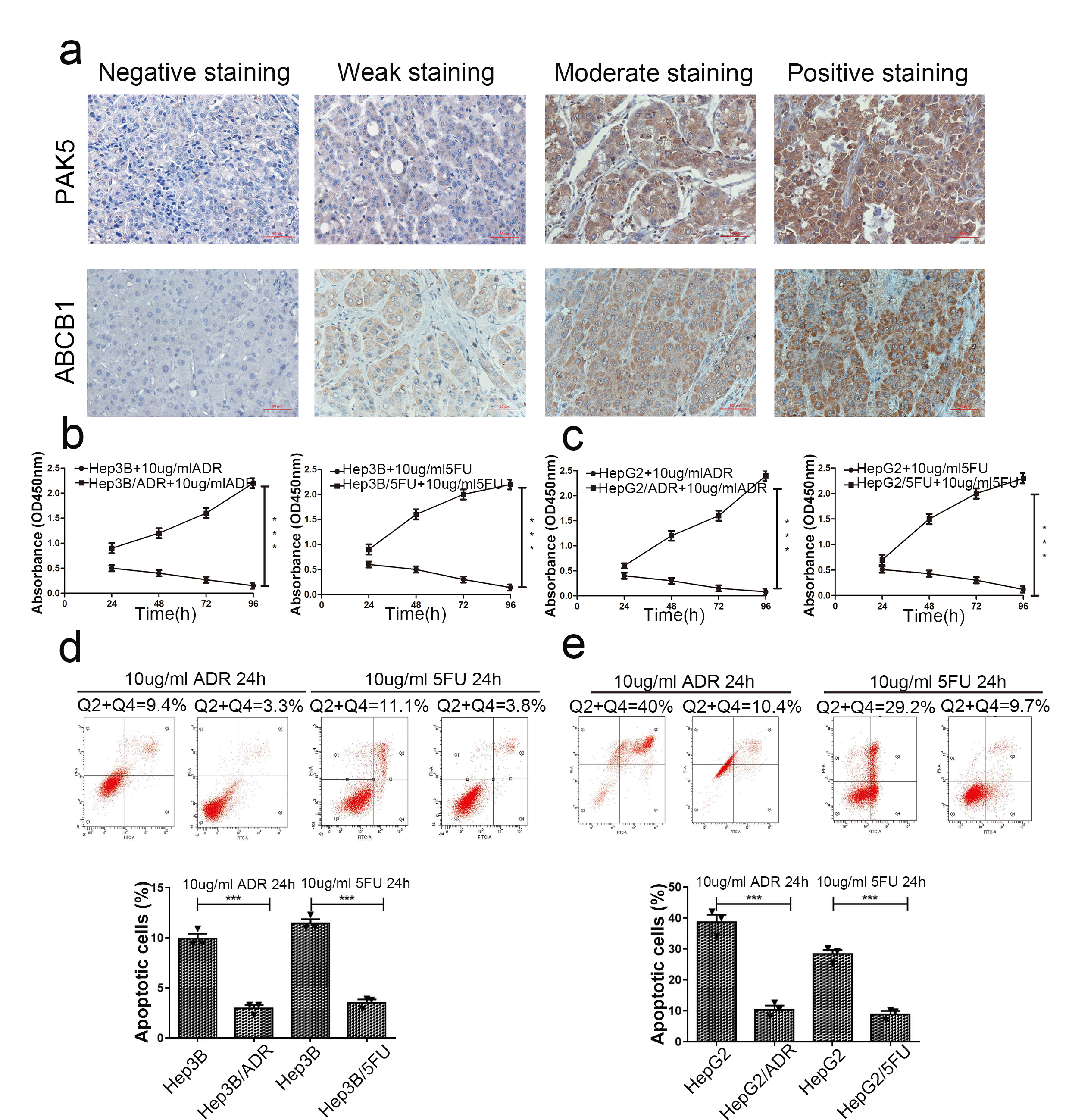

Supplement: Supplementary file 2 — Supplementary Fig. S1 [file 41392_2020_409_MOESM2_ESM.tif]

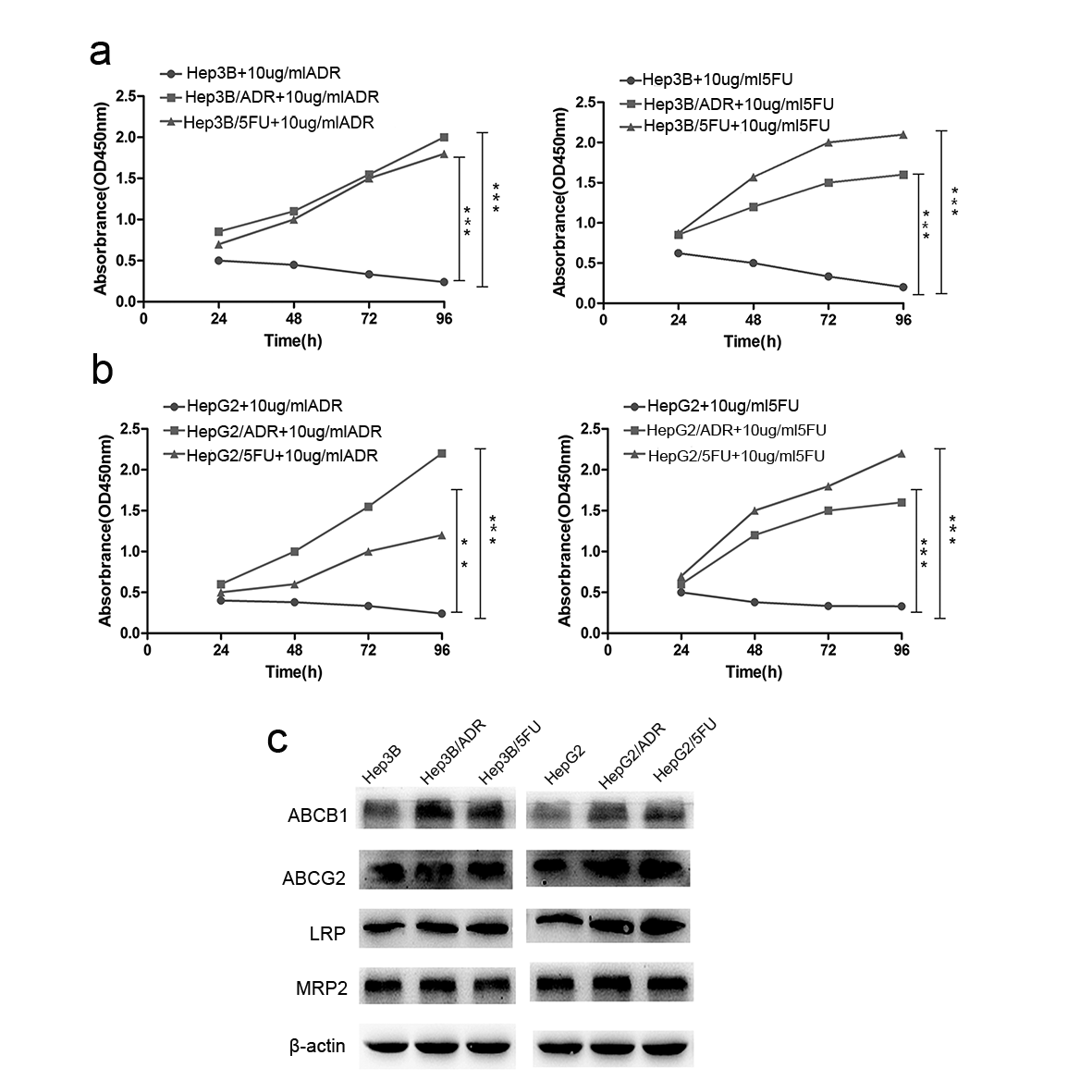

Supplement: Supplementary file 3 — Supplementary Fig. S2 [file 41392_2020_409_MOESM3_ESM.tif]

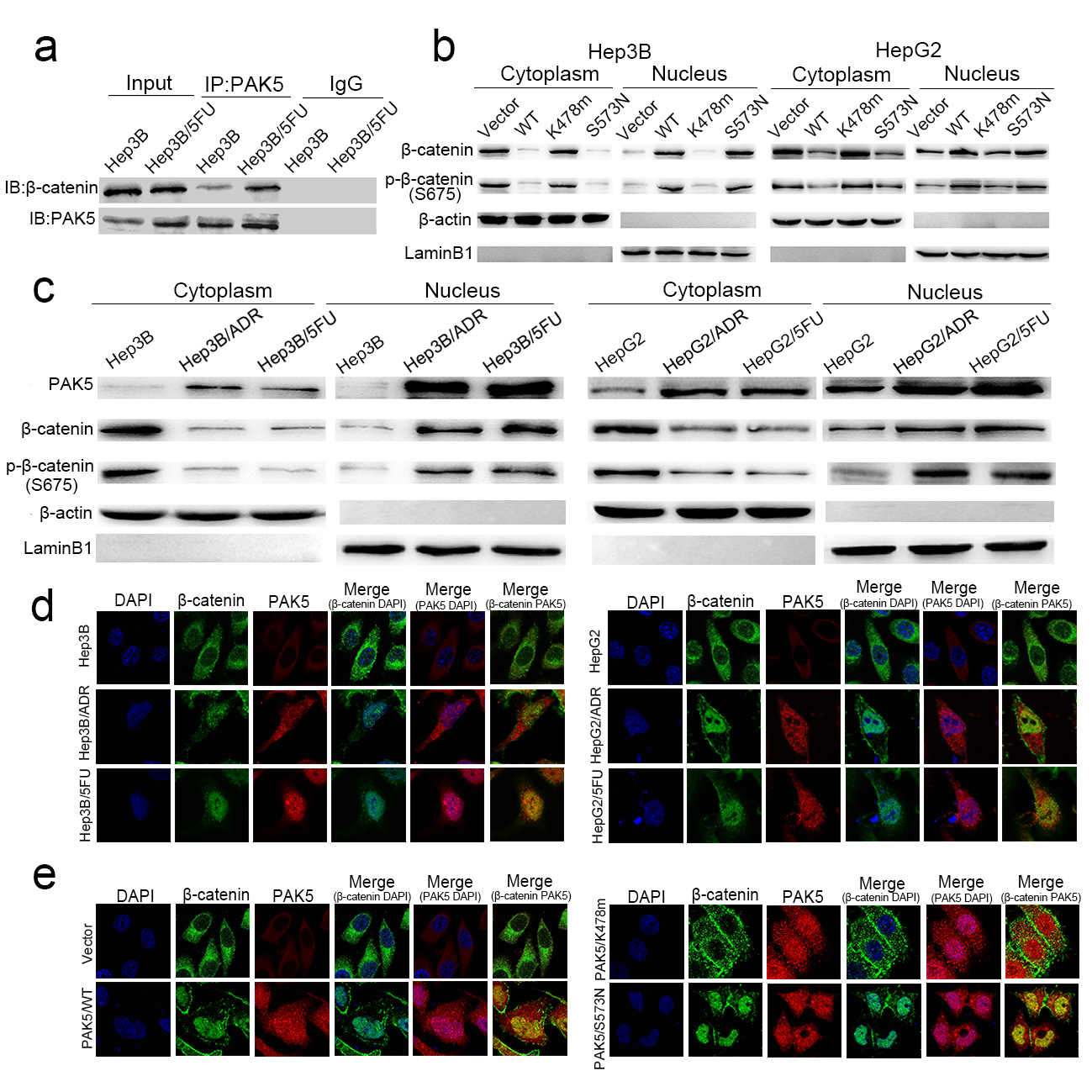

Supplement: Supplementary file 4 — Supplementary Fig. S3 [file 41392_2020_409_MOESM4_ESM.tif]

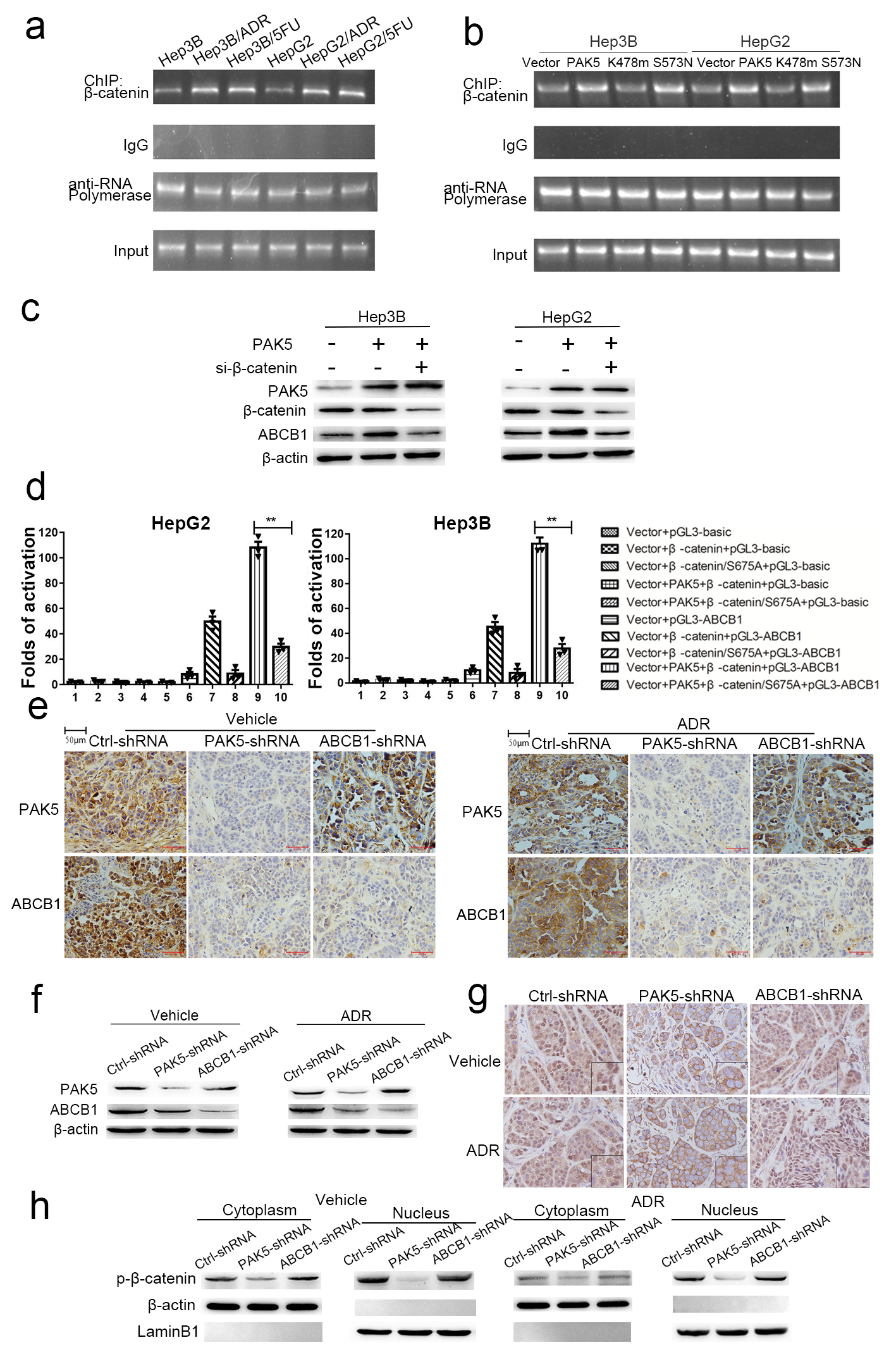

Supplement: Supplementary file 5 — Supplementary Fig. S4 [file 41392_2020_409_MOESM5_ESM.tif]

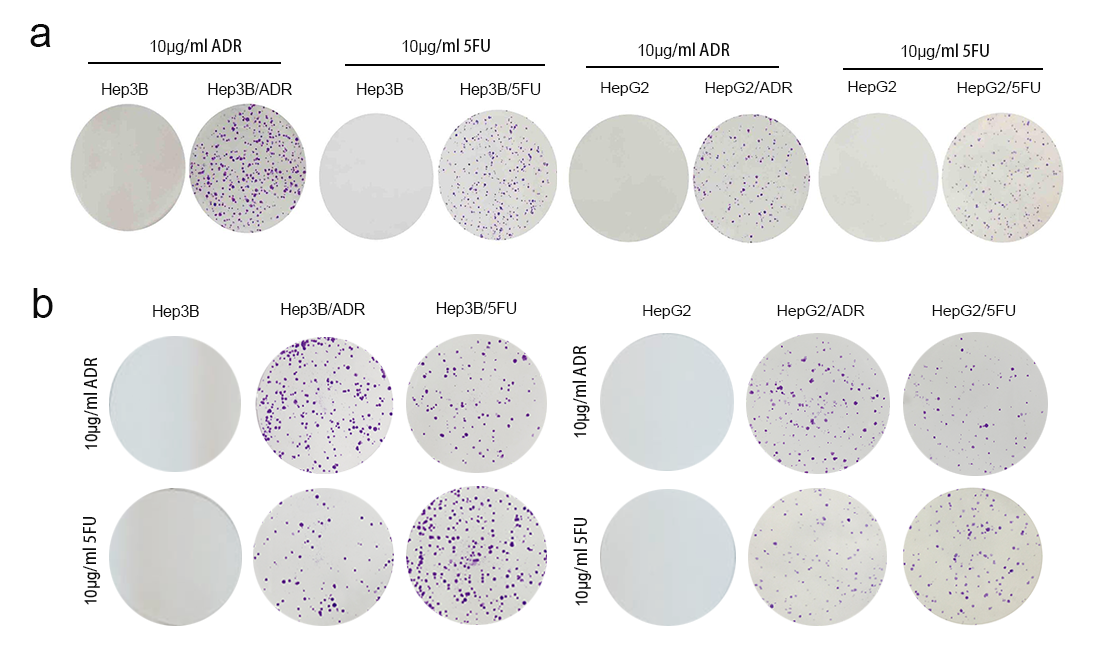

Supplement: Supplementary file 6 — Supplementary Fig. S5 [file 41392_2020_409_MOESM6_ESM.tif]
